# Pre-Axial Fibrolipoma

**Published:** 2013-10-01

**Authors:** Cenk Melikoglu

**Affiliations:** Plastic and Reconstructive Surgeon, Sanliurfa Training and Research Hospital, Turkey.

A baby girl referred from the newborn unit at day 1 of life with a pedunculated soft tissue 3x3.5x 3 cm mass located pre-axially on the palm at the base of her pollex finger. (Fig. 1) The rest of the local and systemic examination was normal. Ultrasonography of the abdomen did not reveal any other congenital anomaly. The lesion was excised. Histopathological examination reported the lesion to be a fibrolipoma. The child was followed up for six months and no signs of recurrence were noted.

**Figure:**
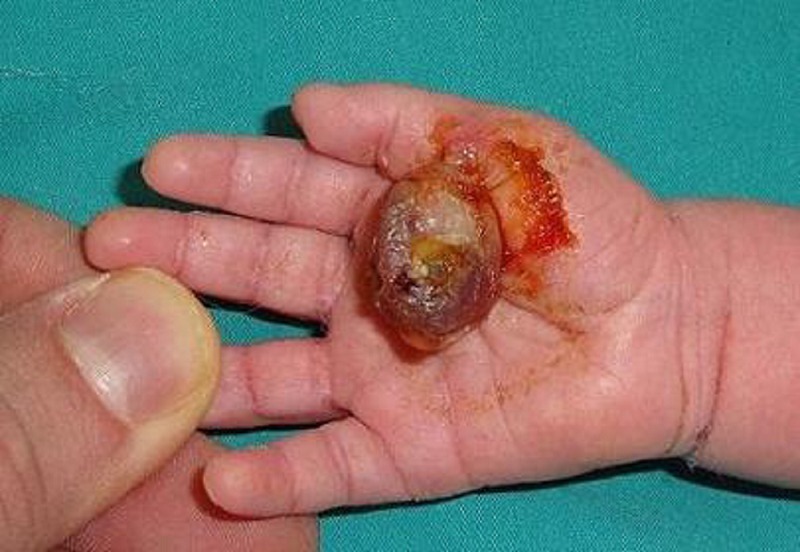
Figure 1: Lesion on palmar aspect of right hand

Fibrolipoma is a benign variant of the family of lipomas which consist of other varieties such as angiolipoma, chondroid lipoma, myolipoma, spindle cell/pleomorphic lipoma, diffuse lipomatous proliferations (lipomatosis) and hipernoma. A pedunculated lesion, as reported here, on the palmar aspect in a newborn is rare and to date no reports have been found to exist in the literature. Other reported congenital sites of occurrence of a fibrolipoma include cornea, anal canal, nasopharynx, mesencephalic protuberation area and the middle ear. The consistency of this lesion varies from soft to firm, depending on the percentage of fibrous tissue and the depth of the tumor. The etiology and possible genetic role in the development of fibrolipoma is still unknown. It is characterised by adipose tissue interposed in a background of fibrous tissue. Treatment of fibrolipoma is entirely surgical and complete excision is the primary treatment of such lesions. [1-3]

## Footnotes

**Source of Support:** Nil

**Conflict of Interest:** None

